# Effect of neoadjuvant therapy on breast cancer biomarker profile

**DOI:** 10.1186/s12885-020-07179-4

**Published:** 2020-07-18

**Authors:** Laura Rey-Vargas, Juan Carlos Mejía-Henao, María Carolina Sanabria-Salas, Silvia J. Serrano-Gomez

**Affiliations:** 1 Grupo de investigación en biología del cáncer, Instituto Nacional de Cancerología, Calle 1a #9-85, Bogotá D. C, Colombia; 2 Pontificia Universidad Javeriana, Bogotá, Colombia; 3 Grupo de patología oncológica, Instituto Nacional de Cancerología, Bogotá, Colombia; 4 Subdirección de Investigaciones - Instituto Nacional de Cancerología de Colombia, Bogotá, Colombia

**Keywords:** Breast neoplasms, Immunohistochemistry, Neoadjuvant therapy, Biomarkers, Heterogeneity

## Abstract

**Background:**

Breast cancer clinical management requires the assessment of hormone receptors (estrogen (ER) and progesterone receptor (PR)), human epidermal growth factor receptor 2 (HER2) and cellular proliferation index Ki67, by immunohistochemistry (IHC), in order to choose and guide therapy according to tumor biology. Many studies have reported contradictory results regarding changes in the biomarker profile after neoadjuvant therapy (NAT). Given its clinical implications for the disease management, we aimed to analyze changes in ER, PR, HER2, and Ki67 expression in paired core-needle biopsies and surgical samples in breast cancer patients that had either been treated or not with NAT.

**Methods:**

We included 139 patients with confirmed diagnosis of invasive ductal breast carcinoma from the Colombian National Cancer Institute. Variation in biomarker profile were assessed according to NAT administration (NAT and no-NAT treated cases) and NAT scheme (hormonal, cytotoxic, cytotoxic + trastuzumab, combined). Chi-squared and Wilcoxon signed-rank test were used to identify changes in biomarker status and percentage expression, respectively, in the corresponding groups.

**Results:**

We did not find any significant variations in biomarker status or expression values in the no-NAT group. In cases previously treated with NAT, we did find a statistically significant decrease in Ki67 (*p < 0.001)* and PR (*p = 0.02605*) expression. When changes were evaluated according to NAT scheme, we found a significant decrease in both Ki67 status (*p = 0.02977*) and its expression values (*p < 0.001*) in cases that received the cytotoxic treatment.

**Conclusions:**

Our results suggest that PR and Ki67 expression can be altered by NAT administration, whereas cases not previously treated with NAT do not present IHC biomarker profile variations. The re-evaluation of these two biomarkers after NAT could provide valuable information regarding treatment response and prognosis for breast cancer patients.

## Background

Breast cancer is the malignancy with the highest incidence (46.3 per 100.000) and mortality rates (13.0 per 100.000) in women worldwide. According to the Surveillance, Epidemiology and End Results Program (SEER), in 2018 breast cancer accounted for 15.3% of all new cancer cases and 6.7% of all cancer deaths in the United States (US) [1, 2].

Neoadjuvant therapy (NAT) has become an important strategy to reduce tumor size in locally advanced breast cancer and facilitate breast conservative surgery, along with monitoring treatment response and eliminating possible micrometastasis [3–5]. In order to choose an appropriate NAT scheme according to tumor biology, a preoperative evaluation on core-needle biopsies from the primary tumor is performed, where histological type and grade are assessed. Additionally, immunohistochemistry (IHC) of biomarkers, such as: hormone receptors (estrogen receptor (ER) and progesterone receptor (PR)), human epidermal growth factor receptor 2 (HER2) and the cellular proliferation index (Ki67), is also analyzed to guide the therapy and predict survival [4, 6, 7].

These biomarkers have been used as surrogates for breast cancer classification into four main intrinsic subtypes: luminal A, luminal B, HER2-enriched and triple negative (TN) [8]. Both, luminal A and luminal B tumors express ER, therefore these patients are candidates for hormone therapy with ER modulators or aromatase inhibitors [8–11], whilst HER2-enriched and TN subtypes lack the expression of hormone receptors, therefore are mainly treated with biological therapy agents such as trastuzumab or pertuzumab, and cytotoxic chemotherapy, respectively [5, 11, 12].

Standard clinical recommendations indicate the assessment of ER, PR, HER2 and Ki67 by IHC in biopsy samples [13, 14]. Nevertheless, many retrospective studies have reported changes in biomarker expression in surgical specimens after NAT administration [15–21]. The main changes correspond to discordances in hormone receptors and HER2 status [22], along with decreases in the percentage of expression, especially for PR and Ki67 [23–26]. Most of these studies end up suggesting the need to re-evaluate its expression, justifying its importance not only to assess tumor response to treatment but to adjust therapy according to these changes [3, 27]. However, other studies show that these changes are not statistically significant [28] and suggest that the re-evaluation of biomarker expression after NAT might not be necessary, especially for health care institutions with limited resources, as it is the case for many hospitals in Latin-America, including Colombia [29].

Given the prognostic value of biomarkers expression and its important role for deciding treatment scheme, the aim of this study was to compare the IHC expression of ER, PR, HER2, and Ki67 in core-needle biopsies and surgical excision specimens in NAT-treated and non-treated breast cancer samples from patients diagnosed in the Colombian National Cancer Institute (NCI), in order to evaluate NAT effect on biomarker expression profile.

## Methods

### Clinical samples and data collection

This is a retrospective study that included 139 breast cancer patients diagnosed with invasive ductal carcinoma (IDC) at the Colombian NCI between 2013 and 2014. Patients were included if they met the following eligibility criteria: 1) histologically confirmed diagnosis of IDC, 2) availability of formalin-fixed paraffin-embedded (FFPE) tissue blocks from mastectomies or breast-conserving surgeries that contained at least 10% of tumor content, 3) availability of IHC slides from core-needle biopsies, and 4) paired IHC biomarker information on biopsy and surgical specimens. Patients with in situ breast carcinoma were excluded. A single pathologist confirmed the histological diagnosis and re-evaluated the expression of the IHC markers from each patient.

This study was approved by the Colombian NCI ethics committee, and according to the Colombian laws, it was considered that no informed consent was required.

Pathology reports were reviewed to obtain information regarding histopathological diagnosis, nodal status, surgical margins, invasion and histological grade. Treatment information was retrieved from clinical records. NAT-treated patients were categorized based on their neoadjuvant scheme in four groups: 1) hormonal, which includes letrozole and/or exemestane, 2) cytotoxic, which includes AC (doxorubicin and cyclophosphamide), taxanes and/or platinums, 3) cytotoxic + trastuzumab, which includes the same therapeutic agents from the cytotoxic scheme plus trastuzumab, and 4) combined, which includes both hormonal and cytotoxic therapeutic agents.

### Immunohistochemistry

IHC for ER, PR, HER2 and Ki67 expression was performed on 3 μm-thick sections from a single FFPE with the highest tumor representation. Staining was carried out using the Roche Benchmark XT automated slide preparation system (Roche Ltd., Switzerland). Positive and negative controls were included and DAB (3,3′ diaminobenzidine) was used as chromogen.

A single pathologist analyzed biomarker expression from surgery blocks and re-evaluated the IHC slides from core-needle biopsies. Hormone receptors (ER and PR) and Ki67 expression values were calculated as the percentage of positive nuclear staining in the IHC slide evaluated. Status of hormone receptors was considered positive when they exceeded 1% of nuclear staining in tumor cells. HER2 was defined as: positive (3+) for complete and intense circumferential membrane within > 10% of tumor cells; ambiguous (2+) for incomplete and/or weak/moderate circumferential membrane staining within > 10% of tumor cells, or complete membrane staining but within ≤10% of tumor cells; negative (1+) for incomplete faint membrane staining within > 10% of tumor cells; and negative (0+) for absence of staining, according to the recommendations of the American Society of Clinical Oncology (ASCO)/College of American Pathologists (CAP) guideline [30]. For analysis purposes, Ki67 was categorized as high (≥20%) or low (< 20%) expression.

### Statistical analysis

We analyzed changes in biomarker status in paired samples from biopsy and surgical specimens, according to NAT administration, using Chi-squared test for categorical variables. Changes in biomarker expression, as continuous variables, were evaluated using the Wilcoxon signed-rank test for paired samples. All analyses were conducted using R software (version 1.2.5033). Differences were considered statistically significant if *p* < 0.05.

## Results

### Clinical-pathological characteristics

Clinical-pathological characteristics of patients included in this study are presented in Table 1. All tumors were classified as IDC, of which the majority presented a clinical stage of III (49.6%) and a Scarff-Bloom-Richardson score of II (59.7%). Sixty-four patients (46%) had positive invasion, from which 38 (45.2%) corresponded to lympho-vascular type. One hundred thirteen (81.3%) of the patients included had axillary lymph node dissection, from which 67 (59.3%) had lymph node involvement. Seventy-eight patients (56.1%) received NAT based mainly in cytotoxic chemotherapy (71.8%).

**Table 1:** Clinical-pathological characteristics of patients at diagnosis

	N (%)
Clinical stage	
I (I, Ia, Ib)	11 (7.9)
II (IIa, IIb)	57 (41.0)
III (IIIa, IIIb, IIIc)	69 (49.6)
IV	2 (1.4)
Scarff-Bloom Richardson	
I	10 (7.2)
II	83 (59.7)
III	46 (33.1)
Invasion	
Yes	64 (46.0)
No	55 (39.6)
Unknown	20 (14.4)
Type of invasion	
Lympho-vascular	38 (45.2)
Dermal	6 (7.1)
Lympho-vascular and perineural	9 (10.7)
Dermal lymphatic	5 (6.0)
Perineural	4 (4.8)
Dermal lymphatic and perineural	1 (1.2)
Dermal and perineural	1 (1.2)
Unknown	20 (23.8)
Axillary lymph node dissection	
Yes	113 (81.3)
No	26 (18.7)
Involvement of lymph nodes	
Yes	67 (59.3)
No	46 (40.7)
NAT administration	
Yes	78 (56.1)
No	61 (43.9)
Type of NAT scheme	
Hormonal	6 (7.7)
Cytotoxic	56 (71.8)
Cytotoxic + trastuzumab	11 (14.1)
Combined (hormonal+ cytotoxic)	5 (6.4)

*NAT* Neoadjuvant therapy

### Biomarker status in core-needle biopsy and surgical excisional specimens

Results from biomarker status in core-needle biopsy showed that most cases were ER positive (80.6%), PR positive (73.4%), HER2 negative (77.0%) and had a high Ki67 proliferation index (≥20%) (66.2%). Biomarker status in surgical specimens presented a similar distribution, where most cases were also ER positive (79.1%), PR positive (71.2%), HER2 negative (71.9%) and had a high Ki67 proliferation index (53.3%). In order to assess the impact of NAT treatment in biomarker status and its expression values, we performed an analysis in paired samples stratified by NAT administration.

### Changes in biomarker status and expression in the no-NAT group

#### Biomarker status

We compared the biomarker status between core-needle biopsy and the surgical specimen in sixty-one cases (43.9%) that did not receive NAT. We neither find statistically significant changes for ER/PR status (*p = 1*) nor for Ki67 (*p = 0.5796*) (Table 2). Even though these are cases that did not receive any type of treatment before surgery that could have affected their tumor biology, interestingly, we observed two cases that changed from PR positive status in biopsy to negative in the surgical specimen; other two cases went from negative status in the biopsy to positive in the surgical specimen for the same biomarker. On the other hand, although not statistically significant, we also observed variations in Ki67 status. From thirty-seven cases with a high Ki67 expression (≥20%) in the biopsy, thirty-two remained in this category, while 4 cases changed its status from high to low Ki67 expression (< 20%) (See Supplementary Table 1, Additional file 1).

**Table 2:** Biomarker status in cases that did and did not receive NAT, in biopsy and surgical specimens

		No-NAT group			NAT group		
		(***N*** = 61)			(***N*** = 78)		
	Categories	Biopsy	Surgery	***p*** value	Biopsy	Surgery	***p*** value
**Estrogen Receptor**	**Positive**	46 (75.4)	46 (75.4)	1	66 (84.6)	64 (82.1)	0.8299
	**Negative**	15 (24.6)	15 (24.6)		12 (15.4)	14 (17.9)	
**Progesterone Receptor**	**Positive**	42 (68.9)	42 (68.9)	1	60 (76.9)	57 (73.1)	0.7115
	**Negative**	19 (31.1)	19 (31.1)		18 (23.1)	21 (26.9)	
**HER2**	**Positive**	9 (14.8)	11 (18.0)	0.4165	10 (12.8)	9 (11.5)	0.6616
	**Negative**	47 (77.0)	41 (67.2)		60 (76.9)	59 (75.6)	
	**Ambiguous**	5 (8.2)	9 (14.8)		7 (9.0)	10 (12.8)	
**Ki67 status**	**Low (< 20%)**	21 (34.4)	23 (37.7)	0.5796	19 (24.4)	41 (52.6)	< 0.001
	**High (≥20%)**	37 (60.7)	37 (60.7)		55 (70.5)	37 (47.4)	
	**Unknown**	3 (4.9)	1 (1.6)		4 (5.1)	0 (0.0)	

*NAT* Neoadjuvant therapy

For HER2 status, we did not find statistically significant changes in paired samples (*p = 0.416*). However, we did observe a modification in the HER2 classification for some cases not previously treated with NAT. Even though an ambiguous status does not correspond to an actual HER2 classification, fluorescence in situ hybridization (FISH) confirmatory results were not available for all cases within this category. Among cases that presented a gain of positive HER2 status, two changed from negative to positive and one changed from ambiguous to positive. Additionally, five cases changed from negative to ambiguous status; from these cases, FISH confirmatory results were available for two of them, with negative result for HER2 amplification in the surgical specimen. The remaining three cases did not have FISH confirmatory results at the surgical sample.

On the other hand, among cases that presented loss of HER2 status, one case changed from positive to ambiguous and another changed from ambiguous to negative status. Both cases had HER2 negative amplification confirmatory results at the surgical sample (Table 3).

**Table 3:** HER2 classification changes in the NAT and no-NAT group from paired biopsy and surgical specimens

	Surgery Biopsy	Negative	Ambiguous	Positive	***p*** value	No. cases with loss of HER2	No. cases with gain of HER2
**No-NAT group (*N*** **= 61)**	Negative	40	5	2	0.4165	2	8
	Ambiguous	1	3	1			
	Positive	0	1	8			
**NAT group (*N*** **= 78)**	Negative	55	4	1	0.6616	4	5
	Ambiguous	2	5	0			
	Positive	2	0	8			
	Unkown	0	1	0			

*NAT* Neoadjuvant therapy

#### Biomarker expression

**Fig. 1:**
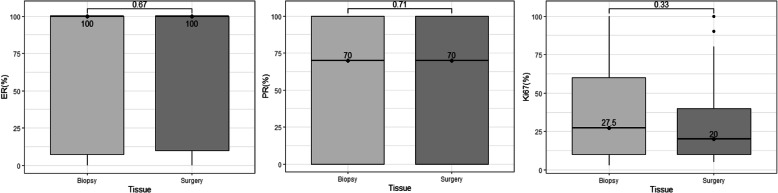
Changes of biomarker expression in biopsy and surgical samples, in the no-NAT group. Points indicate the median value for each measure. ER: Estrogen receptor; PR: Progesterone receptor

We also compared ER, PR and Ki67 expression values between paired specimens. Median values were 100, 70 and 27.5%, respectively, in core-needle biopsies. After surgery, expression values did not show statistically significant changes for any of the three biomarkers (100, 70 and 20%, respectively) (Fig. 1).

### Changes in biomarker status and expression in the NAT group

#### Biomarker status

Seventy-eight cases (56.1%) received NAT. Tumor size measured before and after therapy were compared in these patients. The mean tumor size before NAT were 51.9 mm, and after treatment it significantly decreased to 29.3 mm (*p* < *0.01*).

We did not find any statistically significant changes in ER nor PR status in paired samples in the NAT group (Table 2). Nevertheless, as expected, we observed some cases that did present a modification in their hormone receptor status after NAT. We observed changes from positive to negative status for ER in two cases, one of which also showed loss of PR expression. Four additional cases also changed their PR status from positive to negative. On the other hand, we observed gain of PR status in two cases.

Evaluation of Ki67 status showed statistically significant changes between paired biopsy and surgical specimen (*p* < *0.001*) (Table 2). From fifty-five cases with a high Ki67 expression (≥20%) in the biopsy, thirty-five remained in this category, while twenty cases changed its status from high to low Ki67 expression (< 20%) (See Supplementary Table 1, Additional file 1).

Regarding HER2 status, no statistically significant changes between biopsy and surgical sample were observed (*p = 0.662*), nonetheless, a small number of cases showed changes in HER2 status. Among positive HER2 tumors at biopsy, two cases changed to negative status, and similarly, among cases with ambiguous HER2 status at diagnosis, two cases changed to negative. Lastly, from 60 cases initially defined as HER2 negative in the biopsy, four turned to ambiguous and one case to positive status (Table 3).

Changes in biomarkers status according to NAT scheme was also analyzed. We only found statistically significant variations in Ki67 status between biopsy and surgical specimens in cases treated with the cytotoxic scheme (*p = 0.02977*) (See Supplementary Table 2, Additional file 1). Other findings, although not statistically significant, include a trend for hormone receptors and Ki67 status loss after treatment with the cytotoxic (ER positive: 83.9% vs. 82.1%; PR positive: 76.8% vs. 73.2%) and cytotoxic + trastuzumab schemes (ER positive: 72.7% vs. 63.6%; PR positive: 72.7% vs. 54.5%, High Ki67: 81.8% vs. 54.5%). Interestingly, cases previously treated with cytotoxic therapy presented a trend for a gain of HER2 positive and ambiguous status (HER2 positive: 0% vs 1.8%; HER2 ambiguous: 8.9% vs. 12.5%), whereas in the cytotoxic + trastuzumab scheme group, HER2 showed a tendency for loss of positive status (HER2 positive: 90.9% vs 72.7%).

#### Biomarker expression

**Fig. 2:**
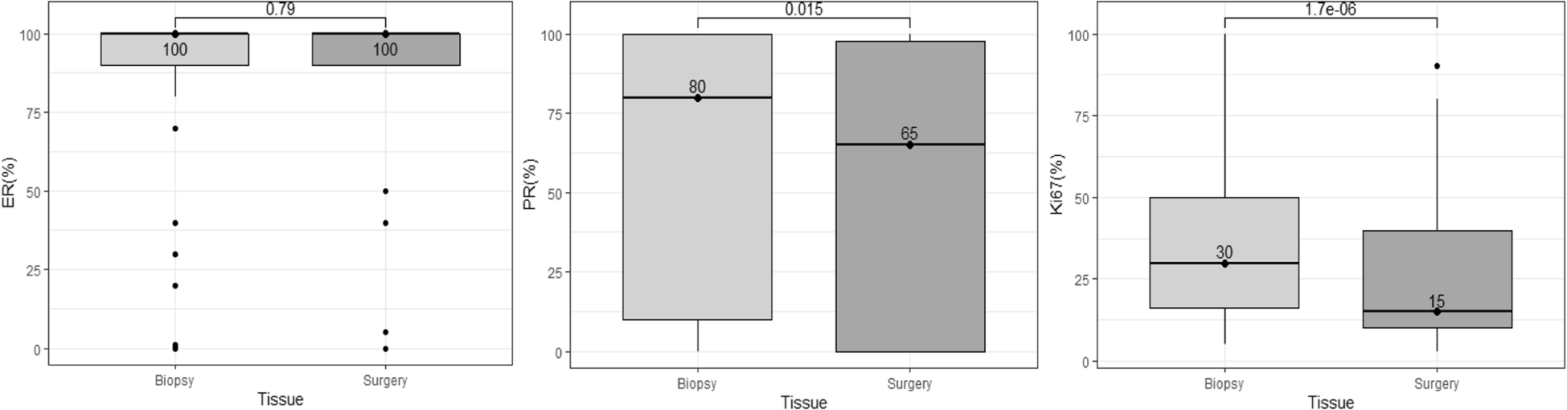
Changes of biomarker expression in biopsy and surgical samples in the NAT group. Points indicate the median value in each measure. ER: Estrogen receptor; PR: Progesterone receptor

The ER, PR and Ki67 median expression values in core-needle biopsy were 100, 80 and 30%, respectively. After surgery, PR and Ki67 expression significantly decreased to 65% (*p = 0.01466)* and 15% (*p* < *0.001*), respectively, whilst ER showed no statistically significant variation **(**Fig. 2). Additionally, we analyzed changes of biomarker expression according to the NAT scheme, and only found a statistically significant decrease for Ki67 expression in cases that received cytotoxic treatment (*p* < *0.001*). Even though not statistically significant, we still could observe a trend for a lower PR expression values in surgical samples for all treatment schemes groups (Hormonal: 100% vs. 80%, Cytotoxic: 80% vs. 70%, Cytotoxic + trastuzumab: 40% vs. 20%, Combined: 90% vs. 20%) (See Supplementary Table 3, Additional file 1).

## Discussion

In the current study, we aimed to analyze changes in IHC biomarker status and expression in paired biopsy and surgical samples in breast cancer cases treated and non-treated with NAT, given that changes in biomarker expression may have several clinical implications for disease outcome in breast cancer patients [31–33]. It may also affect adjuvant therapy regimen, as variation in breast cancer intrinsic subtype after NAT could lead to the addition or discontinuation of therapy schemes [16, 34].

It has been well described that NAT treatment affects Ki67 index, as it targets mainly cycling cells and major cell proliferation pathways [35]. We observed a significant decrease in tumor size and in Ki67 expression values only in the NAT-treated group. Despite the fact that we did not analyze differences in outcome according to these changes, a decrease in Ki67 expression after NAT has been associated with a good clinical-pathological response, better disease-free survival and overall survival [36, 37], whereas no reduction in Ki67 expression after NAT have been associated with a significantly higher risk of breast cancer recurrence and death [38]. Interesting results reported by Dowsett et al. [35] show that Ki67 expression values after 2 weeks of NAT were more useful as prognostic markers for prediction of recurrence-free survival than baseline Ki67 expression before NAT. Our results showing a significant decrease in Ki67 expression after NAT, along with previously reported results, highlight the utility of assessing this biomarker in the surgical specimen after NAT, for prognosis and patients’ clinical outcomes evaluation.

It has been well reported that chemotherapy may have several effects on tumor biology, which could potentially alter biomarker expression [3, 39]. We found a statistically significant decrease in PR expression values between biopsy and surgical specimens after NAT, which is consistent with other reports where the PR, along with the Ki67 index, are the most commonly altered biomarkers after NAT administration [7, 15, 16, 31]. Contrary to what has been reported for Ki67, PR expression loss has been associated with worse tumor characteristics [15] and poor clinical outcomes [23, 32, 40]. It has also been shown that loss of PR expression may be an indicator of a decrease of hormone sensitivity in tumor cells, activation of alternate proliferation pathways such as PI3K/AKT/mTOR [41, 42], and also, the induction of a non-functional ER state which could lead to a diminished response to endocrine therapy, specifically in ER-positive/PR-negative tumors [18, 43].

Loss of ER expression has also been associated with bad clinical outcomes as it affects tumor response to endocrine therapy [44]. Nevertheless, as we reported here, variation in ER expression after NAT is much less frequent than for PR. We did not observe statistically significant changes in ER status nor its expression in neither of the NAT treated or not-treated cases, although we did observe two cases with status loss in the NAT-treated group. These results suggest that analyzing ER after NAT administration may not be as useful as a prognostic marker, as it could be PR and Ki67 status evaluation.

On the other hand, gain of hormone receptor expression after NAT is associated with significantly better outcomes, compared with patients with unchanged hormone receptor expression [45, 46]. It has even been shown that the improvement on survival rates for patients with ER and PR expression gain is dependent on the magnitude of change [47], however, gain of hormone receptor expression is much less frequently reported [22, 31, 32]. In our study, no gain in ER status was observed in neither of the NAT-treated nor non-treated cases, whilst for PR, we observed gain of status in only two cases from the NAT-treated group. Overall, these results suggest that the gain of hormone receptor may not be frequent enough for its implementation to assess treatment response and disease outcomes.

HER2 status variation between biopsy and surgical samples are reported to be less frequent than for hormone receptors and Ki67 index [32, 48]. We did not find statistically significant changes in HER2 biomarker status neither in the NAT-treated nor non-treated cases. Some reports have found important changes in HER2 expression, which seem to be driven not just by NAT, but by specific types of therapeutic agents [33, 40, 49]. Ignatov et al. [25] reported that trastuzumab administration was associated with a decrease in HER2 expression in 47.3% of cases, and interestingly, when pertuzumab was added to the trastuzumab-NAT scheme, the decrease in HER2 expression rise to 63.2%. In our data, when we assessed HER2 variations according to type of NAT regimen, no statistically significant changes were found in neither of the NAT-schemes groups, including the cytotoxic + trastuzumab group. Despite our results not being statistically significant, we did observe some cases in the cytotoxic + trastuzumab scheme group with a decrease in HER2 status. Hypotheses regarding HER2 downregulation after treatment includes the internalization in endosomal compartments and lysosomal degradation of HER2 receptor induced by anti-HER2 agents (pertuzumab, trastuzumab) [50]. Nonetheless, as have been shown, *ERBB2* amplification when evaluated by FISH remains stable after NAT treatment [51].

Undoubtedly, cancer treatments may alter in some degree tumor gene expression [52], leading to possible modification of the IHC biomarker profile. However, tumor heterogeneity is also an important factor to take into account when considering changes in biomarker expression between tumor samples, especially when changes are observed in non-previously NAT treated cases [53], as we reported here. Tumor heterogeneity in breast cancer has been observed in multiple studies [17, 54–56]. Rye et al. [56] evaluated tumor heterogeneity of ER and HER2 expression within individual breast tumors at different time points, and reported the presence of tumor cells within the same sample with both HER2+/ ER+ and HER2+/ER- expression profile, reveling a high rate of cell-to-cell variation. Tumor heterogeneity may have several clinical implications for patient’s outcome. For example, a heterogeneous expression of HER2 copy number in tumors have been reported to be associated with higher risks of relapse and breast cancer death [56]. Such findings are expected given that this kind of intra-tumoral heterogeneity is often the result of clonal evolution, which is highly correlated with metastatic events [57, 58].

External factors different from tumor heterogeneity may also account for changes in biomarkers expression between biopsy and surgical samples in cases not previously treated with NAT [17, 51, 54]. Among these are technical preparation of the IHC stain, fixation times, and inter- and intra-observer variability [17]. It has also been reported that this variability could be a result of the so-called dilution effect, which refers to a decrease of biomarker expression with increasing number of available tumor cells to evaluate at the surgical sample [59]. As have been shown before, larger tumors from surgical excision procedures are more likely to present variations of biomarkers expression between biopsy and surgical samples [15, 55]. This may indicate that at the initial biopsy only a small portion of a tumor is sampled for its evaluation, therefore large tumors could end up being poorly represented and present with IHC profile variations.

Our study certainly had limitations, mainly regarding the small sample size, which limited the statistical power of the analyses, especially when NAT-treated cases were grouped according to the therapy scheme. Small sample size in the hormonal, cytotoxic + trastuzumab and combined NAT-scheme groups may not have allowed us to observe statistically significant expression changes between biopsy and surgical samples. Additionally, all cases were recruited from a single institution and only pathological, but not clinical information was collected. However, our results regarding changes of biomarker expression in NAT-treated and non-treated cases are mostly consistent with what has been reported previously in other studies [17, 51, 54]. On the other hand, we did not have FISH confirmatory amplification results for some cases with HER2 ambiguous result by IHC, which did not allow us to give a more precise classification of these cases. Additionally, it is important to highlight that, unlike most studies evaluating changes of IHC biomarker expression after NAT treatment, we included a group of cases not previously treated with NAT in our analysis, which allowed us to determine if NAT administration could in fact induce changes in the IHC biomarker profile.

## Conclusions

Overall, our results confirmed that NAT administration may cause changes in IHC biomarker profile, mainly in Ki67 and PR expression, and that patients not previously treated with NAT do not present significant changes in biomarker expression. Since it is not cost-effective for the health care system to reassess the expression of each biomarker, for every patient after NAT, we suggest that only PR and Ki67 biomarkers should be reassessed after NAT treatment, as it has been shown that changes in these two may have prognosis implications for breast cancer patients. The implementation of the PR and Ki67 biomarkers as prognosis tools, along with other clinical variables such as tumor stage and nodal status [60, 61], could provide enough information about treatment response and it could be used by physicians to readjust therapy.

## Supplementary information

**Additional file 1: Table S1.** Ki67 classification changes in the NAT and no-NAT group from paired biopsy and surgical specimens. **Table S2.** Biomarker status in biopsy and surgical specimens according to NAT scheme. **Table S3.** Median biomarker expression in biopsy and surgical specimens according to NAT scheme.

## Data Availability

The dataset analyzed during the current study are available from the corresponding author on reasonable request.

## Abbreviations

NAT: Neoadjuvant therapy
IHC: Immunohistochemistry
ER: Estrogen receptor
PR: Progesterone receptor
HER2: Human epidermal growth factor receptor 2
TN: Triple negative
NCI: National Cancer Institute
IDC: Invasive ductal carcinoma
FFPE: Formalin-fixed paraffin-embedded
FISH: Fluorescence in situ hybridization.

## References

[1] Globocan (2018). Breast: Cancer incidence and mortality statistics worldwide and by region.

[2] SEER: Surveillance E and ERP (2018). Female Breast Cancer - Cancer Stat Facts.

[3] Penault-Llorca F, Radosevic-Robin N (2016). Biomarkers of residual disease after neoadjuvant therapy for breast cancer. Nat Rev Clin Oncol.

[4] Untch M, Konecny GE, Paepke S, Von Minckwitz G (2014). Current and future role of neoadjuvant therapy for breast cancer. Breast.

[5] Liu SV, Melstrom L, Yao K, Russell CA, Sener SF (2010). Neoadjuvant therapy for breast cancer. J Surg Oncol.

[6] National Institute for Health and Care Excellence (2018). Early and locally advanced breast cancer: diagnosis and management, NICE guideline.

[7] Lee HC, Ko H, Seol H, Noh DY, Han W, Kim TY (2013). Expression of immunohistochemical markers before and after neoadjuvant chemotherapy in breast carcinoma, and their use as predictors of response. J Breast Cancer.

[8] Prat A, Perou CM (2011). Deconstructing the molecular portraits of breast cancer. Mol Oncol.

[9] Perou CM, Sørlie T, Eisen MB, van de Rijn M, Jeffrey SS, Rees CA (2000). Molecular portraits of human breast tumours. Nature..

[10] Sørlie T, Perou CM, Tibshirani R, Aas T, Geisler S, Johnsen H (2001). Gene expression patterns of breast carcinomas distinguish tumor subclasses with clinical implications. Proc Natl Acad Sci U S A.

[11] Eroles P, Bosch A, Alejandro Pérez-Fidalgo J, Lluch A (2012). Molecular biology in breast cancer: intrinsic subtypes and signaling pathways. Cancer Treat Rev.

[12] Dai X, Li T, Bai Z, Yang Y, Liu X, Zhan J (2015). Breast cancer intrinsic subtype classification , clinical use and future trends. Am J Cancer Res.

[13] Duffy MJ, Harbeck N, Nap M, Molina R, Nicolini A, Senkus E (2017). Clinical use of biomarkers in breast cancer: Updated guidelines from the European Group on Tumor Markers (EGTM). Eur J Cancer Elsevier Ltd.

[14] Provenzano E, Bossuyt V, Viale G, Cameron D, Badve S, Denkert C (2015). Standardization of pathologic evaluation and reporting of postneoadjuvant specimens in clinical trials of breast cancer: recommendations from an international working group. Mod Pathol.

[15] Zhou X, Zhang J, Yun H, Shi R, Wang Y, Wang W (2015). Alterations of biomarker profiles after neoadjuvant chemotherapy in breast cancer : tumor heterogeneity should be taken into consideration. Oncotarget.

[16] Xian Z, Quinones AK, Tozbikian G, Zynger DL (2017). Breast cancer biomarkers before and after neoadjuvant chemotherapy: does repeat testing impact therapeutic management?. Hum Pathol.

[17] Yang YF, Liao YY, Li LQ, Xie SR, Xie YF, Peng NF (2013). Changes in ER, PR and HER2 receptors status after neoadjuvant chemotherapy in breast cancer. Pathol Res Pract.

[18] Neubauer H, Gall C, Vogel U, Hornung R, Wallwiener D, Solomayer E (2008). Changes in tumour biological markers during primary systemic chemotherapy (PST). Anticancer Res.

[19] Gahlaut R, Bennett A, Fatayer H, Dall BJ, Sharma N, Velikova G (2016). Effect of neoadjuvant chemotherapy on breast cancer phenotype, ER/PR and HER2 expression - implications for the practising oncologist. Eur J Cancer.

[20] Jin G, Han Y, Liu C, Chen L, Ding B, Xuan S (2015). Evaluation of biomarker changes after administration of various neoadjuvant chemotherapies in breast cancer. Int J Clin Exp Pathol.

[21] Dede DS, Gumuskaya B, Guler G, Onat D, Altundag K, Ozisik Y (2013). Evaluation of changes of biologic markers ER, PR, HER 2 and Ki-67 in breast cancer with administration of neoadjuvant dose- dense doxorubicin, cyclophosphamide followed by paclitaxel. J BUON.

[22] Peng J, Zhang X, Song J, Ran L, Luo R, Wang Y (2019). Neoadjuvant chemotherapy reduces the expression rates of ER, PR, HER2, Ki67, and P53 of invasive ductal carcinoma. Medicine (Baltimore).

[23] Tural D, Karaca M, Zirtiloglu A, M Hacioglu B, Sendur MA, Ozet A (2019). Receptor discordances after neoadjuvant chemotherapy and their effects on survival. J BUON.

[24] Jin X, Jiang YZ, Chen S, Da Yu K, Shao ZM, Di GH (2015). Prognostic value of receptor conversion after neoadjuvant chemotherapy in breast cancer patients: a prospective observational study. Oncotarget..

[25] Ignatov T, Gorbunow F, Eggemann H, Ortmann O, Ignatov A (2019). Loss of HER2 after HER2-targeted treatment. Breast Cancer Res Treat.

[26] Wu YT, Li X, Lu LJ, Gan L, Dai W, Shi YL (2018). Effect of neoadjuvant chemotherapy on the expression of hormone receptors and Ki67 in Chinese breast cancer patients: a retrospective study of 525 patients. J Biomed Res.

[27] Kang Y-J, Lee H-B, Kim YG, Han J, Kim Y, Yoo T-K (2019). Ki-67 expression is a significant prognostic factor only when progesterone receptor expression is low in estrogen receptor-positive and HER2-negative early breast Cancer. J Oncol.

[28] Kinsella MD, Nassar A, Siddiqui MT, Cohen C (2012). Estrogen receptor (ER), progesterone receptor (PR), and HER2 expression pre- and post- neoadjuvant chemotherapy in primary breast carcinoma: a single institutional experience. Int J Clin Exp Pathol.

[29] Gribble JN, Preston SH, Population NRC (US) C on P, National Academies Press (US) (1993). Health Policy Issues in Three Latin American Countries: Implications of The Epidemiological Transition. The Epidemiological Transition: Policy and Planning Implications for Developing Countries: Workshop Proceedings.

[30] Wolff AC, Hammond MEH, Hicks DG, Dowsett M, McShane LM, Allison KH (2013). Recommendations for human epidermal growth factor receptor 2 testing in breast Cancer: American Society of Clinical Oncology/College of American Pathologists Clinical Practice Guideline Update. J Clin Oncol.

[31] Yang L, Zhong X, Pu T, Qiu Y, Ye F, Bu H (2018). Clinical significance and prognostic value of receptor conversion in hormone receptor positive breast cancers after neoadjuvant chemotherapy. World J Surg Oncol.

[32] Ahn S, Kim HJ, Kim M, Chung YR, Kang E, Kim EK (2018). Negative conversion of progesterone receptor status after primary systemic therapy is associated with poor clinical outcome in patients with breast cancer. Cancer Res Treat.

[33] Shuai Y, Ma L (2019). Prognostic value of pathologic complete response and the alteration of breast cancer immunohistochemical biomarkers after neoadjuvant chemotherapy. Pathol Res Pract.

[34] De La Cruz LM, Harhay MO, Zhang P, Ugras S (2018). Impact of Neoadjuvant Chemotherapy on Breast Cancer Subtype: Does Subtype Change and, if so, How?: IHC Profile and Neoadjuvant Chemotherapy. Ann Surg Oncol.

[35] Dowsett M, Smith IE, Ebbs SR, Dixon JM, Skene A, A’Hern R (2007). Prognostic value of Ki67 expression after short-term presurgical endocrine therapy for primary breast cancer. J Natl Cancer Inst.

[36] Enomoto Y, Morimoto T, Nishimukai A, Higuchi T, Yanai A, Miyagawa Y (2016). Impact of biomarker changes during neoadjuvant chemotherapy for clinical response in patients with residual breast cancers. Int J Clin Oncol.

[37] Penault-Llorca F, Abrial C, Raoelfils I, Chollet P, Cayre A, Mouret-Reynier M (2008). Changes and predictive and prognostic value of the mitotic index, Ki-67, Cyclin D1, and Cyclo-oxygenase-2 in 710 operable breast Cancer patients treated with Neoadjuvant chemotherapy. Oncologist..

[38] Cabrera-Galeana P, Muñoz-Montaño W, Lara-Medina F, Alvarado-Miranda A, Pérez-Sánchez V, Villarreal-Garza C (2018). Ki67 changes identify worse outcomes in residual breast Cancer tumors after Neoadjuvant chemotherapy. Oncologist..

[39] Schmitt MW, Loeb LA, Salk JJ (2016). The influence of subclonal resistance mutations on targeted cancer therapy. Nat Rev Clin Oncol.

[40] Guarneri V, Dieci MV, Barbieri E, Piacentini F, Omarini C, Ficarra G (2013). Loss of HER2 positivity and prognosis after neoadjuvant therapy in HER2-positive breast cancer patients. Ann Oncol.

[41] Brodie A, Sabnis G (2011). Adaptive changes result in activation of alternate signaling pathways and acquisition of resistance to aromatase inhibitors. Clin Cancer Res.

[42] Riggio M, Polo L, Blaustein M, Colman-Lerner A, Lü I, Lanari C (2012). PI3K/AKT pathway regulates phosphorylation of steroid receptors, hormone independence and tumor differentiation in breast cancer. Carcinogenesis..

[43] Cui X, Schiff R, Arpino G, Osborne CK, Lee AV (2005). Biology of progesterone receptor loss in breast cancer and its implications for endocrine therapy. J Clin Oncol.

[44] Li C, Fan H, Xiang Q, Xu L, Zhang Z, Liu Q (2019). Prognostic value of receptor status conversion following neoadjuvant chemotherapy in breast cancer patients: a systematic review and meta-analysis. Breast Cancer Res Treatment.

[45] Lim SK, Lee MH, Park IH, You JY, Nam B-H, Kim BN (2016). Impact of molecular subtype conversion of breast cancers after Neoadjuvant chemotherapy on clinical outcome. Cancer Res Treat.

[46] Tacca O, Penault-Llorca F, Abrial C, Mouret-Reynier M, Raoelfils I, Durando X (2007). Changes in and prognostic value of hormone receptor status in a series of operable breast Cancer patients treated with Neoadjuvant chemotherapy. Oncologist..

[47] Parinyanitikul N, Lei X, Chavez-Macgregor M, Liu S, Mittendorf EA, Litton JK (2015). Receptor status change from primary to residual breast cancer after neoadjuvant chemotherapy and analysis of survival outcomes. Clin Breast Cancer.

[48] Niikura N, Tomotaki A, Miyata H, Iwamoto T, Kawai M, Anan K (2016). Changes in tumor expression of HER2 and hormone receptors status after neoadjuvant chemotherapy in 21 755 patients from the Japanese breast cancer registry. Ann Oncol.

[49] Van de Ven S, Smit VTHBM, Dekker TJA, Nortier JWR, Kroep JR (2011). Discordances in ER, PR and HER2 receptors after neoadjuvant chemotherapy in breast cancer. Cancer Treat Rev.

[50] Hughes JB, Rødland MS, Hasmann M, Madshus IH, Stang E (2012). Pertuzumab increases 17-AAG-induced degradation of ErbB2, and this effect is further increased by combining pertuzumab with trastuzumab. Pharmaceuticals..

[51] Li P, Liu T, Wang Y, Shao S, Zhang W, Lv Y (2013). Influence of Neoadjuvant chemotherapy on HER2/neu status in invasive breast Cancer. Clin Breast Cancer..

[52] Lee SC, Xu X, Lim YW, Lau P, Sukri N, Lim SE (2009). Chemotherapy-induced tumor gene expression changes in human breast cancers. Pharmacogenet Genomics.

[53] Zardavas D, Irrthum A, Swanton C, Piccart M (2015). Clinical management of breast cancer heterogeneity. Nat Rev Clin Oncol.

[54] Mann GB, Fahey VD, Feleppa F, Buchanan MR (2005). Reliance on hormone receptor assays of surgical specimens may compromise outcome in patients with breast cancer. J Clin Oncol.

[55] Cavaliere A, Sidoni A, Scheibel M, Bellezza G, Brachelente G, Vitali R (2005). Biopathologic profile of breast cancer core biopsy: is it always a valid method?. Cancer Lett.

[56] Rye IH, Trinh A, Sætersdal AB, Nebdal D, Lingjærde OC, Almendro V (2018). Intratumor heterogeneity defines treatment-resistant HER2+ breast tumors. Mol Oncol.

[57] Krøigård AB, Larsen MJ, Lænkholm AV, Knoop AS, Jensen JD, Bak M (2015). Clonal expansion and linear genome evolution through breast cancer progression from pre-invasive stages to asynchronous metastasis. Oncotarget..

[58] Barry P, Vatsiou A, Spiteri I, Nichol D, Cresswell GD, Acar A (2018). The spatiotemporal evolution of lymph node spread in early breast cancer. Clin Cancer Res.

[59] Romero Q, Bendahl P-O, Klintman M, Loman N, Ingvar C, Rydén L (2011). Ki67 proliferation in core biopsies versus surgical samples-a model for neo-adjuvant breast cancer studies. BMC Cancer.

[60] Hayashi N, Takahashi Y, Matsuda N, Tsunoda H, Yoshida A, Suzuki K (2018). The prognostic effect of changes in tumor stage and nodal status after Neoadjuvant chemotherapy in each primary breast Cancer subtype. Clin Breast Cancer..

[61] Knutsvik G, Stefansson IM, Aziz S, Arnes J, Eide J, Collett K (2014). Evaluation of Ki67 expression across distinct categories of breast Cancer specimens: a Population-based study of matched surgical specimens, Core needle biopsies and tissue microarrays. PLoS One.

