# Supplementary material for: Effect of neoadjuvant therapy on breast cancer biomarker profile
Source: BMC Cancer. 2020 Jul 18;20:675. doi: 10.1186/s12885-020-07179-4 (PMC7368678; doi:10.1186/s12885-020-07179-4)
Supplement: Supplementary file 1 — Additional file 1: Table S1. Ki67 classification changes in the NAT and no-NAT group from paired biopsy and surgical specimens. Table S2. Biomarker status in biopsy and surgical specimens according to NAT scheme. Table S3. Median biomarker expression in biopsy and surgical specimens according to NAT scheme. [file 12885_2020_7179_MOESM1_ESM.docx]

**Additional file 1**

**Supplementary Table 1** Ki67 classification changes in the NAT and no-NAT group from paired biopsy and surgical specimens

|  | **Surgery  Biopsy** | **High (≥20%)** | **Low (<20%)** | **Unknown** | ***p* value** |
| --- | --- | --- | --- | --- | --- |
| **No-NAT (N= 61)** | High (>20%) | 32 | 4 | 1 | 0.5796 |
|  | Low (<20%) | 4 | 17 | 0 |  |
|  | Unknown | 1 | 2 | 0 |  |
| **NAT (N= 78)** | High (>20%) | 35 | 20 | 0 | <0.001 |
|  | Low (<20%) | 0 | 19 | 0 |  |
|  | Unknown | 2 | 2 | 0 |  |

NAT: Neoadjuvant therapy.

**Supplementary Table 2** Biomarker status in biopsy and surgical specimens according to NAT scheme

| NAT scheme  (N=78) | | Hormonal (N=6) | | | Cytotoxic (N=56) | | | Cytotoxic + trastuzumab (N=11) | | | Combined (N=5) | | |
| --- | --- | --- | --- | --- | --- | --- | --- | --- | --- | --- | --- | --- | --- |
|  | **Categories** | **Biopsy** | **Surgery** | ***p*** | **Biopsy** | **Surgery** | ***p*** | **Biopsy** | **Surgery** | ***p*** | **Biopsy** | **Surgery** | ***p*** |
| ER | Positive | 6 (100.0) | 6 (100.0) | 1 | 47 (83.9) | 46 (82.1) | 1 | 8 (72.7) | 7 (63.6) | 1 | 5 (100.0) | 5 (100.0) | 1 |
|  | Negative | 0 (0.0) | 0 (0.0) |  | 9 (16.1) | 10 (17.9) |  | 3 (27.3) | 4 (36.4) |  | 0 (0.0) | 0 (0.0) |  |
| PR | Positive | 5 (83.3) | 6 (100.0) | 1 | 43 (76.8) | 41 (73.2) | 0.8273 | 8 (72.7) | 6 (54.5) | 0.6576 | 4 (80.0) | 4 (80.0) | 1 |
|  | Negative | 1 (16.7) | 0 (0.0) |  | 13 (23.2) | 15 (26.8) |  | 3 (27.3) | 5 (45.5) |  | 1 (20.0) | 1 (20.0) |  |
| HER2 | Positive | 0 (0.0) | 0 (0.0) | 1 | 0 (0.0) | 1 (1.8) | 0.4906 | 10 (90.9) | 8 (72.7) | 0.3292 | 0 (0.0) | 0 (0.0) | 0.3679 |
|  | Negative | 5 (83.3) | 5 (83.3) |  | 51 (91.1) | 48 (85.7) |  | 0 (0.0) | 2 (18.2) |  | 4 (80.0) | 4 (80.0) |  |
|  | Ambiguous | 1 (16.7) | 1 (16.7) |  | 5 (8.9) | 7 (12.5) |  | 1 (9.1) | 1 (9.1) |  | 0 (0.0) | 1 (20.0) |  |
|  | Unknown | 0 (0.0) | 0 (0.0) |  | 0 (0.0) | 0 (0.0) |  | 0 (0.0) | 0 (0.0) |  | 1 (20.0) | 0 (0.0) |  |
| Ki67 status | Low (<20%) | 3 (50.0) | 6 (100.0) | 0.1824 | 14 (25.0) | 26 (46.4) | 0.02977 | 1 (9.1) | 5 (45.5) | 0.1184 | 1 (20.0) | 4 (80.0) | 0.1496 |
|  | High (≥20%) | 3 (50.0) | 0 (0.0) |  | 40 (71.4) | 30 (53.6) |  | 9 (81.8) | 6 (54.5) |  | 3 (60.0) | 1 (20.0) |  |
|  | Unknown | 0 (0.0) | 0 (0.0) |  | 2 (3.6) | 0 (0.0) |  | 1 (9.1) | 0 (0.0) |  | 1 (20.0) | 0 (0.0) |  |

NAT: Neoadjuvant therapy

**Supplementary Table 3** Median biomarker expression in biopsy and surgical specimens according to NAT scheme

| **NAT scheme**  **(N=78)** | **Hormonal (N=6)** | | | **Cytotoxic (N=56)** | | | **Cytotoxic + trastuzumab (N=11)** | | | **Combined (N=5)** | | |
| --- | --- | --- | --- | --- | --- | --- | --- | --- | --- | --- | --- | --- |
|  | **Biopsy** | **Surgery** | ***p*** | **Biopsy** | **Surgery** | ***p*** | **Biopsy** | **Surgery** | ***p*** | **Biopsy** | **Surgery** | ***p*** |
| **Estrogen Receptor** | 100% | 100% | NA | 100% | 100% | 0.6704 | 80% | 100% | 0.7893 | 100% | 100% | 1 |
| **Progesterone Receptor** | 100% | 80% | 0.1344 | 80% | 70% | 0.2404 | 40% | 20% | 0.1198 | 90% | 20% | 0.1814 |
| **Ki67** | 17.5% | 10% | 0.09751 | 35% | 20% | <0.001 | 50% | 20% | 0.1405 | 25% | 10% | 0.1814 |

NAT: Neoadjuvant therapy; NA: Not able to calculate
